# Long-Term Outcomes of Liver Transplantation in Hepatocellular Carcinoma with Bile Duct Tumor Thrombus: A Comparison with Portal Vein Tumor Thrombus

**DOI:** 10.3390/cancers15174225

**Published:** 2023-08-23

**Authors:** Ji Soo Lee, Jongman Kim, Jinsoo Rhu, Gyu-Seong Choi, Jae-Won Joh

**Affiliations:** 1 Department of Surgery, Kangdong Sacred Heart Hospital, Hallym University College of Medicine, Seoul 05355, Republic of Korea; 191642@kdh.or.kr; 2 Department of Surgery, Samsung Medical Center, Sungkyunkwan University School of Medicine, Seoul 06351, Republic of Korea; jinsoo.rhu@samsung.com (J.R.); gyuseong.choi@samsung.com (G.-S.C.); 3 Department of Surgery, Samsung Changwon Hospital, Sungkyunkwan University School of Medicine, Changwon 51353, Republic of Korea; jw.joh@samsung.com

**Keywords:** bile duct tumor thrombus, portal vein tumor thrombus, liver transplantation

## Abstract

**Simple Summary:**

Liver transplantation is the last chance for patients with hepatocellular carcinoma (HCC) who can no longer be treated. However, not all HCC patients are eligible for a liver transplant. There are several conditions that are eligible for liver transplantation. Among them, portal vein tumor thrombus (PVTT) is treated as a contraindication and bile duct tumor thrombus (BDTT) as an implicit contraindication. However, recently, a study has been published that performed liver transplantation after locoregional treatment in HCC patients with PVTT. We evaluated the long-term clinical significance of liver transplantation in HCC patients with BDTT. We found that BDTT had as poor a disease-free and overall survival after liver transplantation as PVTT. Liver transplantation in HCC patients with BDTT requires a cautious approach and exploration.

**Abstract:**

Liver transplantation (LT) in patients with hepatocellular carcinoma (HCC) with bile duct tumor thrombus (BDTT) remains controversial. This study analyzed the recurrence and overall survival rates through long-term results after LT in HCC patients with BDTT and compared the results after LT in HCC patients with portal vein tumor thrombus (PVTT). We performed a retrospective study of 45 patients with PVTT, 16 patients with BDTT, and 11 patients with coexisting PVTT and BDTT among HCC patients who underwent LT at a single center from 1999 to 2020. The HCC recurrence rates were 40.4% at 1 year, 30.3.3% at 2 years, and 27.6% at 3 years in the PVTT group; 66.7%, 53.3%, and 46.7% in the BDTT group; and 22.2%, 22.2%, and 0% in the coexisting group (*p* = 0.183). Overall patient survival rates were 68.4% at 1 year, 54.3% at 2 years, and 41.7% at 3 years in the PVTT group; 81.3%, 62.5%, and 48.2% in the BDTT group; and 63.6%, 27.3%, and 0% in the coexisting group (*p* = 0.157). In the multivariate analysis, the pre-transplantation model for tumor recurrence after liver transplantation (MoRAL) score and model for end-stage liver disease (MELD) score were found to be independent risk factors for recurrence and survival in all groups. HCC patients with BDTT showed no difference in recurrence and survival compared with HCC patients with PVTT at the long-term follow-up after LT.

## 1. Introduction

Liver cancer was the sixth most commonly diagnosed cancer and the third most common cause of cancer death worldwide in 2020 [1]. Among primary liver cancers, hepatocellular carcinoma (HCC) accounts for 75% to 85% of primary liver cancer. Liver transplantation (LT), which is considered to be the most effective treatment for HCC, follows selection criteria called the Milan Criteria, which are based on the tumor size, the tumor number, vascular invasion, and extrahepatic metastases [2]. Among vascular invasions, portal vein tumor thrombus (PVTT) is commonly found in 10 to 40% of HCC patients and has been considered to be a contraindication for LT because of its poor prognosis [3,4,5]. However, recently, many transplant communities have attempted to expand the conventional criteria and several studies have shown that living donor liver transplantation (LDLT) has an acceptable prognosis in HCC patients with PVTT and that LT is suitable for HCC patients with PVTT [6,7,8,9]. Therefore, when there is no tumor thrombus in the main portal vein, LT is performed in consideration of the tumor biology.

Bile duct tumor thrombus (BDTT) is less common than PVTT, with a reported incidence of 1.2% to 12.9% [10,11,12,13]. BDTT, like PVTT, is considered to be a poor prognostic sign and the presence of BDTT is considered to be advanced HCC. So far, BDTT has not been included in the LT selection criteria and a few cases or studies have led to LT. Like LT trials for HCC patients with PVTT, LT trials for HCC patients with BDTT have also been attempted but were largely limited to single-institution case series, case reports, and systematic reviews [14,15,16]. These studies analyzed LT outcomes in HCC patients with BDTT alone and without other comparators. However, an attempt to expand the conventional criteria for BDTT requires an appropriate comparative and analysis group and—like BDTT—PVTT, classified as advanced HCC, is also included in the criteria, so it is considered to be suitable as a comparison group.

In this study, we analyzed recurrence and overall survival through long-term results after LT in HCC patients with PVTT and BDTT to evaluate whether it was feasible to expand the criteria for LT to HCC patients with BDTT.

## 2. Materials and Methods

### 2.1. Patients and Data Collection

This was a retrospective study using the medical records of patients who underwent LT at Samsung Medical Center (SMC) in Korea from May 1999 to March 2020. All patients enrolled were adults. The right lobe was used for LDLT and the whole liver was used for deceased donor liver transplantation (DDLT). Patients younger than 18 years of age or those with a confirmed tumor invasion to the main vein or tumor thrombosis or extrahepatic metastases were excluded. All HCC patients were preoperatively evaluated to rule out extrahepatic disease, which is unsuitable for LT, by computed tomography (CT), enhanced magnetic resonance imaging (MRI), positron emission tomography (PET)-CT, bone scintigraphy, gastrofiberscopy, colonoscopy, alpha-fetoprotein (AFP) levels, and protein induced by a vitamin K absence or antagonist-II (PIVKA-II).

### 2.2. Diagnosis of PVTT and BDTT

The diagnoses of PVTT and BDTT were based on a postoperative pathologic examination at SMC. Patients who were diagnosed as PVTT and BDTT were classified according to the location of PVTT and BDTT. In general, they are marked in the order of bile duct branches, but here they were classified on the basis of the description of the pathology records at SMC. The classification of PVTT included type Vp2 (tumor thrombus involving the segmental vein, anterior portal vein, or posterior portal vein; the second-order branch) and type Vp3 (tumor thrombus involving the lobar vein, right portal vein, or left portal vein; the first-order branch). The classification of BDTT included type Dp2 (tumor thrombus involving the segmental branch, anterior bile duct, or posterior bile duct; the second-order branch) and Dp3 (tumor thrombus involving the lobar branch, right bile duct, or left bile duct; the first-order branch). In the case of a coexistence, a coexisted group was set and analyzed to compare the characteristics with the BDTT group or PVTT group.

### 2.3. Postoperative Management and Follow-Up

At SMC, immunosuppression was accomplished as per a prior study [17]. Basiliximab (20 mg) was used as an induction agent in all recipients on the LT day and 4 days after LT. Maintenance immunosuppressive therapy consisted of corticosteroids, tacrolimus, and mycophenolate mofetil (MMF). The optimal blood level of tacrolimus was adjusted to maintain a trough plasma concentration of 10 ng/mL during the first month and 5–8 ng/mL after the first month.

After discharge, patients regularly visited the outpatient clinic. These patients were followed from LT until the last follow-up in February 2022 or until patient death. To check for HCC recurrence, AFP and PIVKA-II levels were measured at every outpatient visit and CT or ultrasonography was performed every 3 months for first years. After that, if there was no recurrence, the period was set every 4 months for the first 5 years and every 1 year after 5 years. MRI or PET-CT was performed if recurrence of HCC was suspected.

The primary endpoint of this study was to reveal the long-term survival outcomes and the secondary endpoint was to analyze risk factors associated with the recurrence of HCC.

### 2.4. Statistical Analysis

The results were expressed as percentages and compared using the sample *t*-test and chi-squared test. All continuous numeric variables were reported as a mean with a standard deviation or as a median with a range. Patient overall survival (OS) and disease-free survival (DFS) rates were calculated using the Kaplan–Meier method and compared using the Cox regression analysis. A *p*-value *<* 0.05 was considered to indicate a statistical significance. Analyses were performed using SPSS 27.0 (SPSS, Chicago, IL, USA).

## 3. Results

Among the patients who underwent LT at SMC between May 1999 and March 2020 using the inclusion criteria, 45 patients with PVTT and 16 patients with BDTT were enrolled in this study. Of the 45 patients with PVTT, 30 (66.7%) patients had the Vp2 PVTT type and 15 (33.3%) patients had the Vp3 PVTT type. In the BDTT group, 15 patients (93.8%) had the Dp2 type and 1 patient (6.3%) had the Dp3 type, totaling 16 patients. Common bile duct involvement was not observed in the BDTT group. In the pathological findings, PVTT and BDTT coexisted in 11 cases and these were in the coexisted group.

### 3.1. Baseline Characteristics

The baseline characteristics of three groups are shown in Table 1. Most patients in the groups were male (97.8% vs. 100% vs. 81.8%) and the median age of patients, which differed only in two groups in the ranges 37–72 years and 23–66 years, was 53 years old and 52 years old in the coexisted group (40–72 years). Intrahepatic metastasis was found in 33 (75%) patients in the PVTT group, 10 (62.5%) patients in the BDTT group, and 4 (36.4%) patients in the coexisted group (*p* = 0.017). Prior to LT, the patients received neoadjuvant treatment, transarterial chemoembolization (TACE), liver resection, radiation therapy, radiofrequency ablation (RFA), or intra-arterial chemotherapy, alone or in a combination. The treatment response was 84.5% in the PVTT group, 81.3% in the BDTT group, and 90.9% in the coexisted group, showing complete or partial remission. However, some did not respond to treatment (4.4% vs. 18.8% vs. 0%) and the desease progressed in 5 (11.1%) patients in the PVTT group and 1 (9.1%) patient in the coexisted group.

### 3.2. Surgical Characteristics

The surgical characteristics of the three groups are summarized in Table 2. The preoperative AFP level, PIVKA-II level, model for tumor recurrence after liver transplantation (MoRAL) score, tumor number, tumor differentiation, maximum tumor size, total tumor size, pathological viability, and microvascular invasion (MVI) were not statistically significantly different between the three groups.

### 3.3. Postoperative Characteristics

Table 3 summarizes the postoperative characteristics of the three groups. Acute cellular rejection, mortality, HCC recurrence, and HCC recurrence organs were not statistically significantly different between the three groups.

### 3.4. HCC Recurrence and Patient Survival

**Figure 1:**
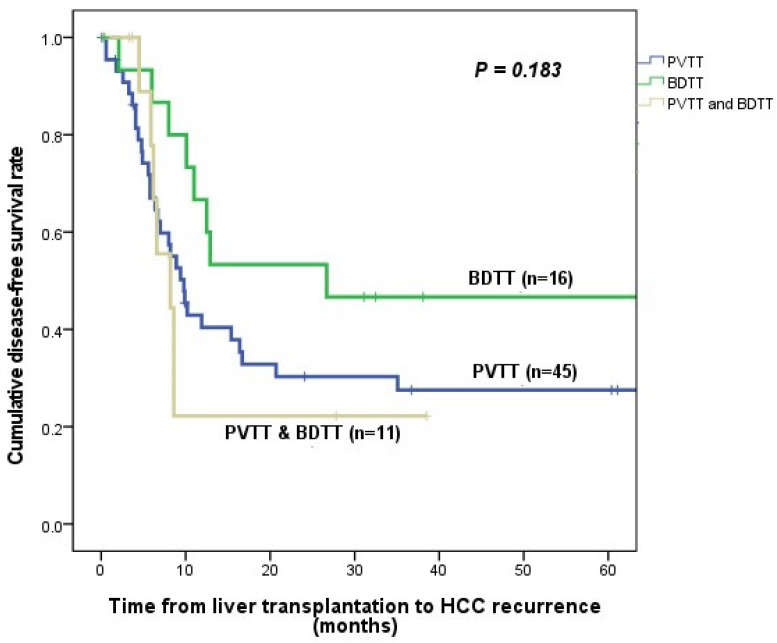
Comparison of disease-free survival curves between portal vein tumor thrombus group, bile duct tumor thrombus group, and coexisted group.

The DFS rates were 40.4% at 1 year, 30.3% at 2 years, and 27.6% at 3 years in the PVTT group; 66.7% at 1 year, 53.3% at 2 years, and 46.7% at 3 years in the BDTT group; and 22.2% at 1 year, 22.2% at 2 years, and 0% at 3 years in the coexisted group. All groups did not differ in DFS (*p* = 0.183; Figure 1).

In the univariate analysis, the MELD score (*p* = 0.006), maximum tumor size (*p* = 0.045), and MoRAL score before LT (*p* < 0.001) were found to be statistically significant risk factors for HCC recurrence. Among them, the MoRAL score before LT (OR 1.001; 95% CI 1.000–1.001; *p* < 0.001) and MELD score (OR 1.041; CI 95% 1.007–1.076; *p* = 0.018) were independent risk factors for DFS (Table 4).

**Figure 2:**
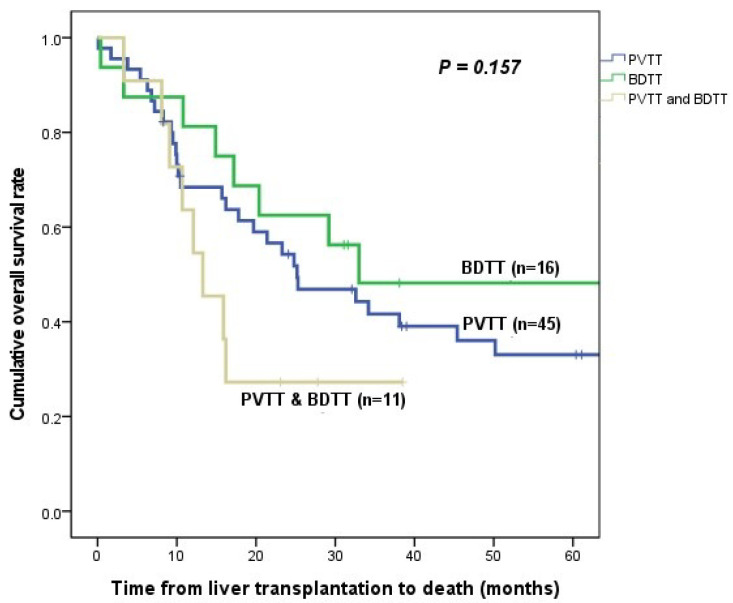
Comparison of overall survival curves between portal vein tumor thrombus group, bile duct tumor thrombus group, and coexisted group.

The OS rates were 68.4% at 1 year, 54.3% at 2 years, and 41.7% at 3 years in the PVTT group; 81.3% at 1 year, 62.5% at 2 years, and 48.2% at 3 years in the BDTT group; and 63.6% at 1 year, 27.3% at 2 years, and 0% at 3 years in the coexisted group. OS rates were also not statistically different between the three groups (*p* = 0.157; Figure 2).

The univariate analysis revealed that the MELD score (*p* = 0.001), Child–Pugh class B (*p* = 0.004) and C (*p* < 0.001), hepatorenal syndrome (HRS) (*p* = 0.004), progressive disease after treatment (PD) (*p* = 0.032), and MoRAL score before LT (*p* = 0.003) were statistically significant risk factors for patient survival. For OS, as with DFS, the MoRAL score before LT (OR 1.001; 95% CI 1.000–1.001; *p* = 0.006) and MELD score (OR 1.105; 95% CI 1.024–1.192; *p* = 0.010) were independent risk factors for patient survival (Table 5).

## 4. Discussion

This study aimed to evaluate the outcomes after LT in HCC patients with BDTT by analyzing the long-term prognosis after LT in HCC patients with PVTT or BDTT. The analysis showed that in both HCC patients with PVTT or BDTT, DFS fell within the first 3 years after transplantation but then stabilized. Similarly, there was a rapid decrease in OS during the first 36 months, but after that, OS in the BDTT group and coexisted group stabilized and OS in the PVTT group continued to decline. OS and DFS stabilized during the first 3 years after LT but all outcomes were poor, at less than 50% during the long-term follow-up. In this study, the OS and DFS rates after LT in HCC patients with BDTT were not different from those after LT in HCC patients with macro- or microvascular invasion and these results were consistent with other analyses [9,18,19,20].

In the results of LT performed in HCC patients with PVTT or BDTT, this long-term follow-up study showed that only the MoRAL score before LT was an independent risk factor affecting both DFS and OS in the three groups. In a recent study, the MoRAL score demonstrated its performance in predicting HCC recurrence after LT [21]. The MoRAL score (11 × √PIVKA-II + 2 × √AFP) uses two tumor markers [22]. The MoRAL scores of the three groups were lower than the 314.8 suggested in studies on MoRAL scores and HCC recurrence [23,24]. Although not statistically significant, the fact that the trends of AFP, PIVKA-II level, and MoRAL scores were higher in the PVTT group than in the BDTT group may have influenced DFS or OS. As such, when considering AFP and PIVKA-II levels before and after LT, and DFS and OS rates between the PVTT group and BDTT group, it was confirmed that the MoRAL score was an independent factor affecting both groups.

In most transplant centers, LT is routinely performed for advanced HCC, but studies of the pathologic findings of BDTT in explanted liver specimens are relatively rare. Thus, this lack of research may lead to a lack of awareness within the transplant community about the impact of BDTT on post-transplant outcomes. Therefore, some studies have reported that surgical resections may increase survival in HCC patients with gross BDTT, although only in highly selected patients [10,25,26]. This situation draws attention to the potential of LT to treat HCC patients with BDTT. However, it has been reported that the recurrence rates and invasiveness of HCC patients with BDTT were significantly higher than those without BDTT [27]. The incidence of macrovascular invasion was found to be 40% in HCC patients with BDTT compared with 15% in HCC patients without BDTT [28]. Several studies have shown that BDTT is characterized by concomitant portal vein invasion in 28.8 to 76.5% of cases [10,25,29]. Zeng et al., reported histological and ultrastructural findings of the mechanism of tumor thrombus formation [30]. The bile duct and portal vein are within the Glissonian sheath together, allowing the tumor to invade adjacent structures. This explains the high prevalent correlation between BDTT and PVTT. Therefore, when HCC patients with BDTT undergo LT, portal vein invasion can lead to HCC recurrence and post-transplant death. In this study as well, the 1-year DFS was 66.7% but at 3 years it dropped to less than 50%. Most of the premature deaths seen in patients undergoing liver transplantation are related to disease recurrence and almost all patients who recurred in the PVTT and BDTT groups died in this study. Therefore, careful decisions are needed for LT in HCC patients with BDTT.

This study was limited by several factors. First, it was a retrospective single-center study and there may have been heterogeneity between the patient groups. The distributions of each patient were different when the tumor thrombus was divided into the branch between the three groups. There was heterogeneity, such as different follow-up time points among patients, different treatment methods for pre-transplant HCC, and an absence of data regarding viral hepatitis history. Second, the long sample inclusion period could lead to bias in surgeons and treatment methods. However, as cases such as PVTT or BDTT are contraindicated for liver transplantation, our cases were bound to be retrospectively identified over a long period of time and the protocols for post-transplantation management have changed little over 20 years.

We also believe that the sample size of BDTT was too small and that a larger number of BDTT samples would have made a clearer comparison with PVTT. However, the reason for such a small number of specimens was that this study was based on the pathological findings of explanted liver from liver transplant cases performed for about 20 years at a single center and liver transplantation was not performed because BDTT was not an indication for liver transplantation. Therefore, in the case of BDTT, which is contraindicated in liver transplantation, a multi-center study is needed to increase the number of samples and such a study may be a step toward new guidelines.

## 5. Conclusions

The results of this study showed that in HCC patients with BDTT, LT was associated with a high risk of HCC recurrence and poor survival at the long-term follow-up, similar to HCC patients with PVTT. LT in HCC patients with BDTT requires a more cautious approach than LT in HCC patients with PVTT. Therefore, LT in HCC patients with BDTT deserves further multi-center long-term studies and exploration.

## Figures and Tables

**Table 1:** Baseline characteristics of portal vein tumor thrombus group, bile duct tumor thrombus group, and coexisted group.

	PVTT (n = 45)	BDTT (n = 16)	Coexisted (n = 11)	*p*-Value
Sex, male	44 (97.8%)	16 (100%)	9 (81.8%)	0.057
Age, years	53 (37–72)	53 (23–66)	52 (40–72)	0.996
BMI	24.8 (17.1–32.9)	23.9 (18.5–27.0)	23.6 (19.1–37.1)	0.164
Hypertension	7 (15.6%)	3 (18.8%)	1 (9.1%)	0.725
Diabetes mellitus	14 (31.1%)	6 (37.5%)	4 (36.4%)	0.657
Hepatic encephalopathy	2 (3.4%)	3 (18.8%)	0 (0%)	0.823
Varix bleeding	3 (5.1%)	0 (0%)	0 (0%)	0.213
Ascites				0.331
None Diuretics—controlled Diuretics—uncontrolled	26 (57.6%) 14 (28.8%) 5 (13.6%)	7 (43.8%) 4 (25.0%) 5 (31.3%)	6 (54.5%) 3 (27.3%) 2 (18.2%)	
ABO incompatibility	9 (12.5%)	0 (0.0%)	2 (18.2%)	0.430
HBV	41 (91.9%)	11 (68.8%)	11 (100%)	0.905
HCV	5 (8.1%)	1 (6.3%)	0 (0%)	0.218
Alcoholic	1 (2.2%)	2 (12.5%)	1 (9.1%)	0.195
NASH	0 (0%)	2 (12.5%)	0 (0%)	0.367
Child–Pugh class				0.138
A B C	21 (46.7%) 17 (37.8%) 7 (15.6%)	6 (37.5%) 5 (31.3%) 5 (31.3%)	4 (36.4%) 2 (18.2%) 5 (45.5%)	
MELD score	10 (7–40)	10 (0–40)	13 (8–40)	0.220
HRS				0.444
None Without CRRT CRRT or HD	42 (93.3%) 2 (4.4%) 1 (1.4%)	15 (93.8%) 0 (0%) 1 (6.3%)	9 (81.8%) 2 (18.2%) 0 (0%)	
SBP	4 (5.6%)	1 (6.3%)	0 (0%)	0.311
Intrahepatic metastasis	33 (75%)	10 (62.5%)	4 (36.4%)	0.017
Neoadjuvant treatments	32 (71.1%)	12 (75.0%)	8 (72.7%)	0.845
TACE	28 (62.2%)	12 (75.0%)	8 (72.7%)	0.216
Liver resection	7 (15.6%)	3 (18.8%)	1 (9.1%)	0.725
Radiation therapy	10 (23.7%)	5 (31.3%)	4 (36.4%)	0.268
RFA	9 (20%)	5 (31.3%)	3 (27.3%)	0.362
Intra-arterial chemotherapy	0 (0%)	0 (0%)	1 (9.1%)	0.048
Treatment response				0.449
CR PR SD PD	35 (77.8%) 3 (6.7%) 2 (4.4%) 5 (11.1%)	11 (68.8%) 2 (12.5%) 3 (18.8%) 0 (0%)	7 (63.6%) 3 (27.3%) 0 (0%) 1 (9.1%)	
Donor type, LDLT	40 (88.9%)	15 (93.8%)	7 (63.6%)	0.091
Donor sex, male	17 (37.8%)	10 (62.5%)	3 (27.3%)	0.958
Donor age, years	32 (17–61)	27 (19–72)	44 (16–82)	0.195
Donor BMI	23.4 (18.7–32.2)	23.2 (18.4–27.1)	21.6 (18.8–32.5)	0.580
Donor hospitalization	10 (6–20)	11 (6–28)	9 (7–15)	0.208

PVTT: portal vein tumor thrombus; BDTT: bile duct tumor thrombus; BMI: body mass index; HBV: hepatitis B virus; HCV: hepatitis C virus; NASH: non-alcoholic steatohepatitis; MELD: model for end-stage liver disease; HRS: hepatorenal syndrome; CRRT: continuous renal replacement therapy; HD: hemodialysis; SBP: spontaneous bacterial peritonitis; TACE: transarterial chemoembolization; RFA: radiofrequency ablation; CR: complete remission; PR: partial remission; SD: stable disease; PD: progressive disease; LDLT: living donor liver transplantation.

**Table 2:** Surgical characteristics of portal vein tumor thrombus group, bile duct tumor thrombus group, and coexisted group.

	PVTT (n = 45)	BDTT (n = 16)	Coexisted (n = 11)	*p*-Value
Preoperative AFP, ng/mL	45.3 (2.2–17,342.0)	18.3 (4.7–7933)	40.3 (3.9–37,478.0)	0.462
Preoperative PIVKA-II, ng/mL	473 (15–16,650)	52 (26–500)	132 (16–520)	0.079
MoRAL score at diagnosis time	336.2 (45.6–1504.1)	83.7 (64.9–424.1)	133.2 (53.8–447.1)	0.079
MoRAL score at LT	273.2 (48.9–3279.1)	161.5 (69.2–1478.5)	107.9 (54.8–458.3)	0.201
MoRAL score, maximum	291.3 (63.9–3017.9)	137.3 (73.3–1684.1)	140.7 (75.1–765.9)	0.111
Tumor number, n				0.189
Solitary	9	9	7	
Multiple	33 (2–50)	7 (2–10)	4 (2–13)	
Tumor differentiation				0.551
Moderate	34 (75.6%)	13 (81.3%)	9 (81.8%)	
Poor	11 (24.4%)	3 (18.8%)	2 (18.2%)	
Maximum tumor size, cm	4.3 (1.0–17.0)	3.8 (1.0–16.0)	4.8 (2–14)	0.838
Total tumor size, cm	10.0 (2.0–29.0)	10.0 (3.0–31.0)	9.6 (3.0–30.0)	0.962
Pathologic viability	11 (24.4%)	5 (31.3%)	4 (36.4%)	0.391
MVI	44 (97.8%)	14 (87.5%)	44 (100%)	0.743
GRWR	0.98 (0.62–2.21)	1.08 (0.81–1.69)	0.95 (0.76–2.29)	0.280
Donor operation time, min	327 (210–545)	347 (203–469)	336 (211–373)	0.805
Recipient operation time, min	526 (247–758)	511 (358–715)	366 (264–752)	0.180
Cold ischemic time, min	87 (37–448)	85 (66–141)	91 (58–415)	0.789
Warm ischemic time, min	36 (13–88)	36 (18–90)	40 (17–60)	0.779
Macrosteatosis, %	5 (1–25)	5 (1–60)	5 (1–25)	0.238
Microsteatosis, %	5 (1–20)	5 (1–25)	10 (1–75)	0.355

PVTT: portal vein tumor thrombus; BDTT: bile duct tumor thrombus; AFP: alpha-fetoprotein; PIVKA-II: protein induced by vitamin K absence or antagonist-II; MoRAL: model for tumor recurrence after liver transplantation, MoRAL score = (11 × $\sqrt{PIVKA-II}$ + 2 × $\sqrt{AFP}$); LT: liver transplantation; MVI: microvascular invasion; GRWR: graft-to-recipient weight ratio.

**Table 3:** Postoperative characteristics of portal vein tumor thrombus group, bile duct tumor thrombus group, and coexisted group.

	PVTT (n = 45)	BDTT (n = 16)	Coexisted (n = 11)	*p*-Value
mTOR inhibitor				0.879
None Combined tacrolimus Only mTOR inhibitor use	14 (31.1%) 29 (64.4%) 2 (4.4%)	7 (43.8%) 6 (37.5%) 3 (18.8%)	4 (36.4%) 6 (54.5%) 1 (9.1%)	
Acute cellular rejection	10 (22.2%)	0 (0%)	2 (18.2%)	0.325
Frequency of acute rejection				0.158
1 time 2 times 3 times	7 2 1	0 0 0	2 0 0	
HCC recurrence	31 (68.9%)	8 (50.0%)	7 (63.6%)	0.456
Mortality	30 (66.7%)	8 (50.0%)	8 (72.7%)	0.928
HCC recurrence organ				0.062
Liver Lung Bone Lymph node Others	10 (22.2%) 18 (40%) 3 (6.7%) 0 (0%) 10 (22.2%)	6 (75.0%) 1 (12.5%) 0 (0%) 1 (12.5%) 0 (0%)	4 (57.1%) 1 (14.3%) 0 (0%) 0 (0%) 2 (28.6%)	
ICU stay before LT, days	0 (0–8)	0 (0–2)	0 (0–3)	0.530
ICU stay after LT, days	6 (3–11)	6 (3–20)	5 (3–29)	0.676

PVTT: portal vein tumor thrombus; BDTT: bile duct tumor thrombus; mTOR: mammalian target of rapamycin; LT: liver transplantation; HCC: hepatocellular carcinoma; ICU: intensive care unit.

**Table 4:** Univariate and multivariate analyses of risk factors for disease-free survival between portal vein tumor thrombus group, bile duct tumor thrombus group, and coexisted group.

	Univariate Analysis			Multivariate Analysis		
	OR	95% CI	*p*-Value	OR	95% CI	*p*-Value
Sex, female	2.490	0.750–8.270	0.136			
HBV	1.125	0.476–2.658	0.788			
MELD score	1.046	1.013–1.080	0.006	1.041	1.007–1.076	0.018
Maximum tumor size	1.082	1.002–1.169	0.045			
Total tumor size	1.038	0.997–1.081	0.067			
Pathologic viability	0.547	0.271–1.103	0.092			
PVTT	1	1	0.197			
BDTT	0.704	0.380–1.305	0.265			
BDTT only	0.519	0.238–1.130	0.099			
PVTT and BDTT	1.187	0.519–2.715	0.685			
Intrahepatic metastasis	1.414	0.754–2.654	0.280			
Locoregional treatments	1.170	0.615–2.225	0.632			
Treatment response						
CR PR SD PD	1 0.506 0.170 1.972	1 0.180–1.422 0.023–1.242 0.766–5.076	0.067 0.196 0.081 0.159			
MoRAL score before LT	1.001	1.001–1.001	<0.001	1.001	1.000–1.001	<0.001

OR: odds ratio; CI: confidence interval; HBV: hepatitis B virus; MELD: model for end-stage liver disease; PVTT: portal vein tumor thrombus; BDTT: bile duct tumor thrombus; CR: complete remission; PR: partial remission; SD: stable disease; PD: progressive disease; MoRAL: model for tumor recurrence after liver transplantation, MoRAL score = (11 × $\sqrt{PIVKA-II}$ + 2 × $\sqrt{AFP}$); LT: liver transplantation.

**Table 5:** Univariate and multivariate analyses of risk factors for overall survival between portal vein tumor thrombus group, bile duct tumor thrombus group, and coexisted group.

	Univariate Analysis			Multivariate Analysis		
	OR	95% CI	*p*-Value	OR	95% CI	*p*-Value
Age	1.018	0.982–1.056	0.338			
Sex, female	2.568	0.773–8.528	0.124			
HBV	1.143	0.451–2.896	0.777			
MELD score	1.055	1.023–1.088	0.001	1.105	1.024–1.192	0.010
Child–Pugh class						
A B C	1 2.935 4.441	1 1.419–6.074 2.063–9.563	<0.001 0.004 <0.001			
HRS, HD, or CRRT	9.076	2.032–40.548	0.004			
Maximum tumor size	1.074	0.992–1.164	0.079			
PVTT	1	1	0.168			
BDTT	0.922	0.502–1.694	0.794			
BDTT only	0.640	0.293–1.398	0.263			
PVTT and BDTT	1.674	0.754–3.720	0.206			
Treatment response						
CR PR SD PD	1 0.473 0.721 2.616	1 0.145–1.544 0.221–2.350 1.087–6.298	0.064 0.215 0.587 0.032			
MoRAL score before LT	1.001	1.000–1.001	0.003	1.001	1.000–1.001	0.006

OR: odds ratio; CI: confidence interval; HBV: hepatitis B virus; MELD: model for end-stage liver disease; HRS: hepatorenal syndrome; HD: hemodialysis; CRRT: continuous renal replacement therapy; BDTT: bile duct tumor thrombus; CR: complete remission; PR: partial remission; SD: stable disease; PD: progressive disease; MoRAL: model for tumor recurrence after liver transplantation, MoRAL score = (11 × $\sqrt{PIVKA-II}$ + 2 × $\sqrt{AFP}$); LT: liver transplantation.

## Data Availability

The datasets generated and analyzed during the current study are not publicly available because the hospital is not permitted to remove the datasets, but they are available from the corresponding author upon reasonable request.
